# Neuron-targeted caveolin-1 overexpression attenuates cognitive loss and pathological transcriptome changes in symptomatic Alzheimer’s disease models

**DOI:** 10.1038/s41392-025-02258-z

**Published:** 2025-05-28

**Authors:** Dongsheng Wang, Andrei V. Chernov, Ryan Lam, Hongxia Wang, Wenxi Li, Xiaojing Li, Tiffany Duong, Shanshan Wang, Brian P. Head

**Affiliations:** 1 Department of Anesthesiology, University of California, San Diego, La Jolla, CA 92093 USA; 2 VA San Diego Healthcare System, San Diego, CA 92161 USA

**Keywords:** Neurological disorders, Gene therapy

## Abstract

Alzheimer’s disease (AD) is a devastating neurodegenerative disorder characterized by progressive synaptic loss and cognitive decline. Gene therapy that augments intrinsic neuroprotective pathways offers a promising strategy to mitigate neurodegeneration and prevent further cognitive loss. Caveolin-1 (Cav-1), a membrane lipid raft (MLR) scaffolding protein, regulates multiple pro-growth and pro-survival signaling pathways within plasmalemmal microdomains. Previously, we showed that AAV9-Synapsin-promoted Cav-1 (*SynCav1*) delivered to presymptomatic AD mice preserved cognitive functions and MLR-associated neurotrophic signaling. However, the therapeutic potential of *SynCav1* delivered at the symptomatic stage of AD had not been tested. Therefore, the current study investigated the effect of hippocampal *SynCav1* delivery at symptomatic age in two distinct preclinical AD models of amyloid pathology: PSAPP and APPKI mice. Our results demonstrated that *SynCav1* delivery to PSAPP and APPKI mice at symptomatic age consistently preserved hippocampal-dependent memory. Transcriptome profiling revealed that PSAPP-*SynCav1* mice exhibited a similar transcript profile to age-matched wild-type mice. Gene Ontology enrichment analysis indicated downregulation of neurodegeneration-specific pathways and upregulation of synaptic and cognitive-related pathways in PSAPP-*SynCav1* mice. In vitro, *SynCav1*-transfected mouse primary cortical neurons exhibited increased p-CaMKII and p-CREB expression, suggesting that *SynCav1* may protect the CNS by enhancing neuronal and synaptic activity. Furthermore, activity-dependent neuroprotective protein (ADNP) was identified as a potential candidate mediating *SynCav1*’s neuroprotective effects on cognition. Subcellular membrane fractionation revealed that *SynCav1* preserved MLR-localized pituitary adenylate cyclase-activating polypeptide type I receptor (PAC1R), a well-known regulator of ADNP expression. Together, these findings highlight *SynCav1* as a unique and promising gene therapy candidate in the treatment of AD.

## Introduction

Alzheimer’s disease (AD) is a devastating neurodegenerative disorder characterized by a progressive decline in cognitive function, including learning, memory, and language processing. Recent data estimate that over 416 million individuals worldwide are on the AD continuum (AD dementia, prodromal AD, and preclinical AD),^1^ a number that is expected to nearly double every 20 years. Given this rapidly growing epidemic patient demographic, there is an urgent need for therapies that can slow or halt AD progression. Extracellular amyloid-beta (Aβ) plaques and intraneuronal neurofibrillary tangles (NFTs) are two pathological hallmarks present in AD-affected brains, both of which cause significant neuronal damage and contribute to cognitive impairment. Currently, there are only a few FDA-approved anti-amyloid monoclonal antibodies (Lecanemab and Donanemab) for early AD treatment. Mechanistically, Lecanemab selectively targets highly toxic soluble Aβ aggregates (Aβ oligomers and protofibrils) to prevent plaque formation and facilitate clearance,^2^ whereas Donanemab binds to and removes existing mature plaques.^3^ While both therapies demonstrated efficacy in decreasing amyloid burden and relieving early symptoms, they produce only moderate effects on disease progression, suggesting that targeting Aβ alone is not sufficient to address the progressive nature of AD. While amyloid pathology is one important aspect of AD, the pathophysiology of AD contains disruption in a broad range of cellular function including synaptic dysfunction, mitochondrial abnormalities, neuroinflammation, and loss of trophic support. In tandem with the rapid development of pharmacological interventions, a significant investment has thus been made toward developing alternative approaches such as neuroprotective gene/cell therapy and anti-inflammatory therapy. Considering the multifaceted nature of AD pathology, a “cocktail” approach, which entails a combination of therapeutic strategies, may prove essential. Thus, genetic interventions that augment neuroprotective transcripts and pro-survival signaling pathways may offer a promising strategy for treating AD.

Caveolin-1 (Cav-1) is a cholesterol-binding and scaffolding protein involved in the formation of membrane/lipid rafts (MLRs), cholesterol-enriched microdomains that are essential for maintaining cell membrane integrity and coordinating signal transduction.^4,5^ Multiple studies have shown that Cav-1 knockdown in vitro results in multiple cellular dysfunctions, including impaired synaptic activity, vesicular trafficking, *N*-methyl-D-aspartate receptor (NMDAR) MLR localization, and mitochondrial homeostasis.^6,7^ In vivo, loss of Cav-1 resulted in disruption in cellular processes involved in synaptic plasticity, axonal growth, and motor function development.^8,9^ Importantly, clinical evidence observed decreased transcript of Cav-1 and Cav-1-associated signaling complexes (neurotransmitter receptors, adenylyl cyclase) in degenerating neurons from CTE patients.^10^ Loss of Cav-1 expression and associated altered subcellular localization have also been implicated in accelerated aging^11,12^ and in neurodegenerative diseases such as Amyotrophic Lateral Sclerosis (ALS).^13^ Previously, we showed decreased Cav-1 expression in the hippocampus of early symptomatic APP/PSEN1 (PSAPP) mice.^14^ Considering the role of Cav-1 in regulating synaptic function and signal transduction, it is possible that decreased neuronal Cav-1 will further exacerbate already impaired cellular function within the degenerating CNS. Thus, neuron-targeted overexpression of Cav-1 presents promising therapeutic potential in the treatment of neurodegenerative diseases like AD.

In the past few years, our group has conducted a serial of studies to explore role of Cav-1 in the neuronal system. Our results showed that neuron-targeted overexpression of Cav-1 in primary neurons significantly enhanced MLR-localized NMDAR, α-amino-3-hydroxy-5-methyl-4-isoxazolepropionic acid receptor (AMPAR), and tyrosine receptor kinase receptors (TrkR) expression, receptor-mediated cAMP formation, and dendritic arborization.^15^ In vivo overexpression of neuronal Cav-1 in the hippocampus showed strong and durable pro-cognitive effects in both adult and aged wild-type (WT) mice.^16^ In the PSAPP mouse model of AD, hippocampal delivery of *AAV9*-Synapsin-Cav-1 (*SynCav1*) at presymptomatic age (3 months) significantly attenuated cognitive deficits, prevented neurodegenerative-related synaptic loss, and maintained mitochondrial dynamics and function at symptomatic stages.^14,17^ While these studies demonstrate the neuroprotective effects of *SynCav1* when delivered to healthy animals and pre-symptomatic AD animals, the therapeutic effect of *SynCav1* treatment at the symptomatic stage of AD remains to be explored.

To address this critical knowledge gap, the primary aim of the current study was to first investigate the therapeutic effect of hippocampal *SynCav1* delivery to symptomatic PSAPP mice. To further explore the applicability of *SynCav1* therapy in AD, we also examined the effects of *SynCav1* hippocampal delivery in a recently developed AD mouse model carrying three human APP mutations, termed *App*^*NL*−*G*−*F*/*NL*−*G*−*F*^ (APPKI), at symptomatic age. Compared to the widely used PSAPP mouse model that features overexpression of APP and overproduction of amyloid-β, the APPKI mouse model more closely recapitulates human AD pathology as it expresses physiological level of APP and exhibits aberrant APP processing seen in human patients.^18^ Utilizing multiple behavioral tests, we assessed the safety and efficacy of *SynCav1* therapy on general behavior and cognitive function. Combining transcriptional analysis and biochemical analysis of subcellular components, we aim to understand the underlying transcriptomic changes and identify biochemical signaling events through which *SynCav1* affords its neuroprotective effects.

## Results

### *SynCav1* gene delivery to early symptomatic PSAPP mice (6 m) preserved hippocampal-dependent learning and memory

**Fig. 1:**
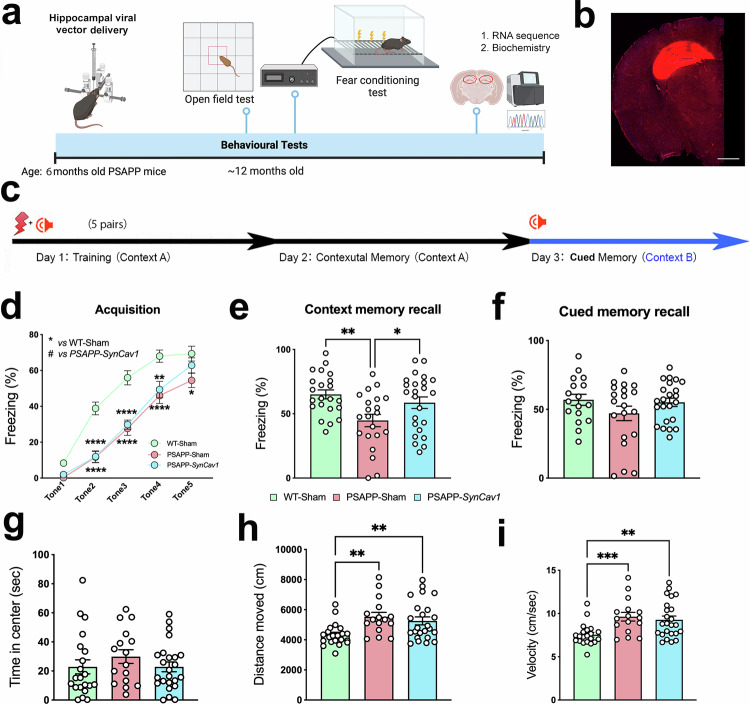
Direct hippocampal delivery of *SynCav1* at symptomatic stage protects learning and memory in PSAPP mice. **a** Schematic of the experimental design. **b** Representative immunofluorescence image of Cav-1 (red) from *SynCav1*-injected brain. Scale bar = 1000 µm. **c** Schematic for classical fear conditioning protocol. **d–f** Assessment of fear memory acquisition, contextual memory recall, and cued memory recall performed at 12 m (6 m post *SynCav1* delivery). Data are presented as percent (%) freezing mean ± SEM. Data were analyzed using two-way analysis of variance (ANOVA) followed by Fisher’s LSD multiple comparisons tests (Day 1) or One-way ANOVA (Day 2 and Day 3). *n* = ~20 per group. **g–i** Assessment of general motor function and anxiety-like behavior by open field test at 12 m. Analysis of time spent in the center area, total distance moved, and velocity. Data were presented as mean ± SEM and analyzed using One-way ANOVA. *n* = ~20 per group. Significance was assumed when *p* < 0.05. **p < 0.01, ***p < 0.005

To evaluate whether *SynCav1* delivery at the symptomatic stage of AD attenuates cognitive deficits, the FC test was conducted at 12 m to assess learning and memory (Fig. 1a–c). Given our previous findings demonstrating that *SynCav1* improves memory recall in normal WT mice,^16^ age and sex-matched WT-*SynCav1* groups were omitted from the present study to reduce experimental redundancy. As shown in Fig. 1d, the WT-Sham group exhibited a rapid acquisition of freezing behavior over the first four tones on Day 1, ultimately reaching a plateau by tone 5. In comparison, both PSAPP-Sham and PSAPP-*SynCav1* groups showed significantly fewer freezing behavior during the first four tones. Notably, at tone five, while PSAPP-*SynCav1* group displayed a similar percent freezing time compared to the WT-Sham group (62.87% vs. 69.39%; *p*-value = 0.17), the PSAPP-Sham group still exhibited significantly fewer freezing behavior versus WT (54.47% vs. 69.39%; *p*-value < 0.01), indicating impaired acquisition ability in the 12 m PSAPP-Sham group. On Day 2, while PSAPP-Sham mice exhibited a significantly decreased percent freezing time versus WT-Sham mice (44.77% vs. 65.07%; *p*-value = 0.001), PSAPP-*SynCav1* mice displayed a percent freezing similar to WT-Sham mice (58.60% vs. 65.07%; *p*-value = 0.28), indicating preserved hippocampal-dependent contextual memory recall (Fig. 1e). Assessment of cue-dependent memory recall on Day 3 revealed that both PSAPP-Sham and PSAPP-*SynCav1* mice exhibited a decreasing trend in freezing behavior compared to WT-Sham mice. No significant difference was detected between PSAPP-*SynCav1* and PSAPP-Sham groups (Fig. 1f).

OF test was also performed to assess locomotion and anxiety-like behavior (Fig. 1g–i). No significant difference in center dwelling time was detected among the three groups. Analysis of velocity and moving distance showed that both PSAPP-Sham and PSAPP-*SynCav1* mice exhibited hyperlocomotion compared to WT-Sham mice, albeit no difference was observed between the two AD groups. These data indicate *SynCav1* hippocampal delivery was not accompanied by adverse side effects related to anxiety or general locomotion.

### *SynCav1* preserved a WT-like transcriptome profile in PSAPP mice

**Fig. 2:**
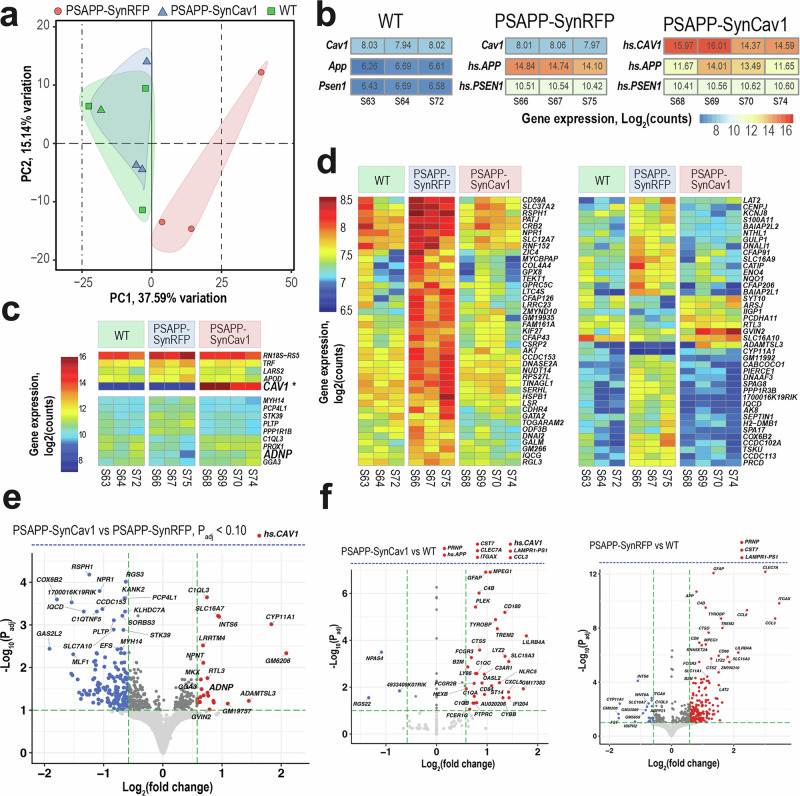
Transcriptome changes in PSAPP mice received *SynCav1*. **a** Principal Component Analysis of normalized gene counts following variance stabilizing transformation in PSAPP-*SynRFP* (red circles), PSAPP-*SynCav1* (blue triangles), and WT (green squares) samples (WT and PSAPP-*SynRFP* groups: *n* = 3; PSAPP-*SynCav1* group: *n* = 4). Colored areas indicate cluster overlaps. **b** Confirmation of phenotypic identity of mice in PSAPP-*SynRFP*, PSAPP-*SynCav1*, and WT groups by raw reads mapping to the human CAV-1 (hs.CAV1, GenBank ID 857), amyloid beta precursor protein (hs.APP, GenBank ID 351) and presenilin 1 (hs.PSEN1, GenBank ID 5663) mRNA sequences (WT and PSAPP-*SynRFP* groups: *n* = 3; PSAPP-*SynCav1* group: *n* = 4). Mouse-specific orthologous genes are indicated as Cav1, App, and Psen1. Colors correspond to Log2(counts) according to scales. **c**, **d** Gene expression heatmaps of significant DEGs. Highly expressed (**c**) and moderately expressed (**d**) DEGs. Colors correspond to Log2(counts) for each sample according to scales. **e**, **f** Volcano scatter plots (-log_10_*P*adj vs log_2_(fold change (FC)) of significant DEGs in PSAPP-*SynCav1* versus PSAPP-*SynRFP* (**e**), PSAPP-*SynCav1* versus WT and PSAPP-*SynRFP* versus WT (**f**). Red and blue colors indicate up- and down-regulated DEGs, respectively. Significance threshold *P*adj <0.1 (-log10(*P*adj) > 1.0) is indicated by a horizontal green dashed line. For data visualization, the arbitrary *post hoc* fold-change thresholds (log_2_FC > 0.58 or log_2_FC < −0.58) were applied as indicated by vertical green dashed lines. DEGs outside the diagram’s scale are indicated by auxiliary axes (horizontal blue dashed lines)

Given the robust neuroprotective effect of *SynCav1* on hippocampal-dependent functions observed in PSAPP mice, bulk RNA-seq was performed to further dissect the effect of *SynCav1* on the transcriptomic profile of PSAPP mice. Principal component (PC) analysis determined that the AD (PSAPP)-*SynRFP* group distinctly separated from the WT control group, indicating a unique global gene expression profile that reflects the underlying AD pathology. In contrast, transcriptional profiles of AD-*SynCav1* (i.e., PSAPP-*SynCav1*) and WT mice demonstrated a substantial overlap in the PC1/PC2 dimensions that accounted for 37.6% and 15.1% total gene expression variation, respectively (Fig. 2a). This overlay indicates a normalization of the gene expression patterns towards a non-diseased state. The phenotypic identity of mice in AD-*SynRFP*, AD-*SynCav1*, and WT groups was confirmed by mapping raw reads to the human CAV1 (GenBank ID 857), amyloid beta precursor protein (APP, GenBank ID 351) and presenilin 1 (PSEN1, GenBank ID 5663) mRNA sequences (Fig. 2b). Hierarchical clustering analysis of the top genes ranked by decreasing variance uncovered clusters of distinctly expressed and co-expressed genes across groups and biological replicates (Fig. 2c, d). Overexpression of Cav-1 was listed in the top genes as expected.

Differentially expressed genes (DEGs) were determined using thresholds of *P*adj <0.1. (supplementary Table S2). Accordingly, in the AD-*SynCav1* vs. AD-*SynRFP* comparison group, 133 and 337 DEGs were estimated to have higher and lower expression, respectively (Fig. 2e). 61 and 4 DEGs exhibited higher and lower expression, respectively, in AD-*SynCav1* compared to WT group (Fig. 2f). Comparison between AD-*SynRFP* and WT groups revealed 377 upregulated and 109 downregulated genes, respectively.

### PSAPP mice exhibit altered neuronal/synaptic signatures that were restored with *SynCav1*

**Fig. 3:**
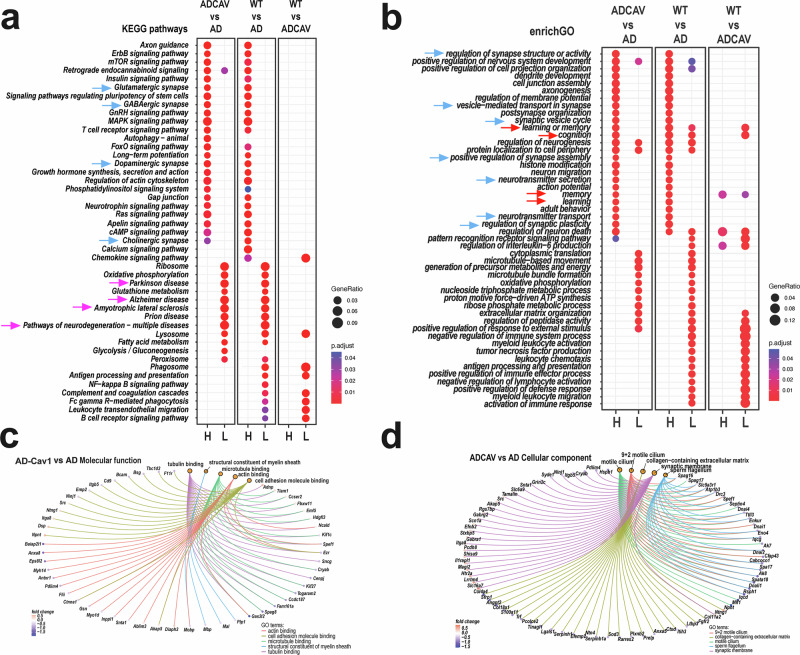
Exploratory gene set enrichment analysis predicted that *SynCav1* upregulated cognitive-related pathways and downregulated neurodegenerative disease-related pathways. **a** The top KEGG pathways were identified by enrichment analysis of significant DEGs (*P* < 0.05, |log_2_FC | >0.58) in AD-Cav1(*SynCav1*) vs AD, WT vs AD, and WT vs AD-Cav1 groups. The complete list is included in Supplementary Data S3. Circles indicate the gene ratios (circle size) and Fisher exact test *P* (*P*adj <0.05, blue to red colors – lower to higher significance) according to the scales. H/L columns: pathways are predicted to have higher (H) or lower (L) activity relative to the comparison group. **b** The top GO Biological Processes (BP) were identified by enrichment analysis of significant DEGs (*P* < 0.05, (|log_2_FC | >0.58) in AD-Cav1 vs AD, WT vs AD, and WT vs AD-Cav1 groups. The complete list is included in Supplementary Data S4. Circles indicate the gene ratios (circle size) and Fisher exact test P (*P*adj <0.05, blue to red colors – lower to higher significance) according to the scales. H/L columns: BP is predicted to have higher (H) or lower (L) activity relative to the comparison group. DEGs specific to the top 5 Molecular Functions (**c**) GO terms and Cellular Component (**d**) Go terms identified by the clusterProfiler enrichment analysis based on lists of significant DEGs (*P* < 0.05, |log_2_FC | >0.58) in AD-Cav1 vs AD groups (AD: *n* = 3; AD-Cav1: *n* = 4). Orange circles indicate GO terms. Color circles indicate DEGs: blue to red - lower to higher log2FC according to respective scales. Color connecting lines link DEGs to respective GO terms

Protein-coding and non-coding RNA (ncRNAs) DEGs annotated in the GO database and fitting the standard statistical criteria (*P* < 0.05, |log2FC | > 0.58) in each WT vs. AD-*SynRFP*, AD-*SynCav1* vs. AD-*SynRFP* and AD-*SynCav1* vs. WT comparison groups were used for enrichment analyses in clusterProfiler.^19^ Signaling pathways from Kyoto Encyclopedia of Genes and Genomes (KEGG) and GO biological processes (BP) terms were ranked based on false discovery rate estimations (Q-values < 0.05), and selected relevant pathways are shown (Fig. 3). The list of predicted GO and KEGG pathways is included in Supplementary Tables S3 and S4. KEGG pathway analysis among 3 groups revealed that multiple dysregulated pathways in AD were reversed in the *SynCav1*-treated group (Fig. 3a). Interestingly, several genes implicated in neurodegenerative disease pathways, including AD (mmu05010), Huntington’s Disease (mmu05016), Parkinson’s Disease (mmu05012), ALS (mmu05014), pathways of neurodegeneration - multiple diseases (mmu05022) were significantly upregulated in AD compared to WT (pink arrows). Conversely, the AD-*SynCav1* group suggested significant downregulation of these neurodegenerative disease pathways. A similar trend was observed in pathways related to synaptic function, as multiple dysregulated pathways involving glutamatergic synapses (mmu04724), GABAergic synapses (mmu04727), dopaminergic synapses (mmu04728), serotonin synapse6) (mmu04724, and cholinergic synapses (mmu04725) in AD were reversed in the AD-*SynCav1* group (blue arrows).

As shown in Fig. 3b, GO biological processes analysis indicated that the AD-*SynCav1* group exhibits higher levels of “learning or memory” (GO:0007611), “Cognition” (GO:0050890), “learning” (GO:0007612), and “memory” (GO:0007613) (red arrows). Furthermore, the AD-*SynCav1* group also exhibits higher levels of synaptic-related pathways (blue arrows), including “synaptic structure and activity” (GO:0050803), “synaptic vesicle cycle” (GO:0099504), “neurotransmitter secretion” (GO:0007269), etc. Since synaptic loss is one of the characteristic anatomical hallmarks that correlate with cognitive deficits in AD patients, these results suggest that neuroprotective effects afforded by *SynCav1* may occur by preserving or augmenting functional synaptic plasticity. As shown in Fig. 3c, tubulin binding, structural constituent of myelin sheath, microtubule binding, cell adhesion molecule binding, and actin binding were identified as the top 5 GO molecular functions. Synaptic membrane, sperm flagellum, motile cilium, 9 + 2 motile cilium, and collagen−containing extracellular matrix were identified as the top 5 GO cellular components (Fig. 3d).

### *SynCav1* gene delivery to mid-symptomatic APPKI mice preserved learning and memory

**Fig. 4:**
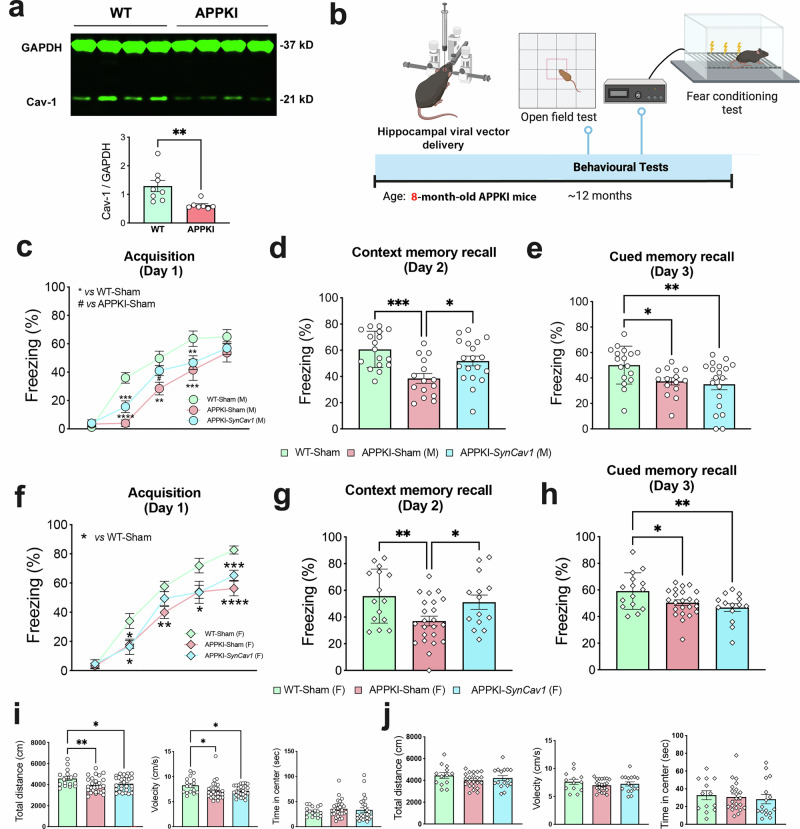
*SynCav1* delivery at mid-symptomatic stage protects hippocampal-dependent memory in APPKI mice. **a** IB of hippocampal homogenates showed significantly decreased Cav-1 expression in 8 m APPKI mice. Data (*n* = 7–8) are presented as mean ± SEM. Data were analyzed using the Student’s *t* test. **b** Experimental schematic for *SynCav1* delivery at 8 m and behavioral tests performed at 12 m. **c–h** FC test evaluates fear memory acquisition, contextual memory recall, and cue-dependent memory recall in male (**c–e**) and female (**f–h**) APPKI mice. Data are presented as percent (%) freezing mean ± SEM. Data were analyzed using two-way analysis of variance (ANOVA) followed by Fisher’s LSD multiple comparisons tests (Day 1) or One-way ANOVA (Day 2 and Day 3). *n* = ~15 for male, *n* = ~20 for female. General motor function and anxiety-like behavior were assessed by OF test in males (**i**) and females (**j**). Analysis of time spent in the center area (left panels), total distance moved (middle panels), and velocity (right panels). Data were analyzed using One-way ANOVA. *n* = ~20. Significance was assumed when *p* < 0.05. **p < 0.01, ***p < 0.005

To further establish the applicability of *SynCav1* therapy in the treatment of AD, we tested the effect of *SynCav1* in a more clinically relevant APP knock-in mouse model that expresses physiological level of humanized mutant APP. Interestingly, a significant decrease in Cav-1 protein expression was detected in hippocampal tissue from APPKI mice at 8 m (Fig. 4a). We thus delivered *SynCav1* to 8-month-old APPKI mice, which already exhibit a wide spectrum of AD-associated phenotypes, including memory deficit, amyloid-β deposits, and widespread neuroinflammation,^20^ and conducted behavior tests at 12 months of age (Fig. 4b).

On Day 1 of FC, both APPKI-Sham mice and APPKI-*SynCav1* mice showed significantly lower percent freezing time versus WT-Sham mice, suggesting a temporal limitation to the neuroprotective effects of *SynCav1* on hippocampus-dependent associative learning in this AD model (Fig. 4c). On Day 2, a significant decrease in freezing behavior was detected in APPKI-Sham mice versus WT-Sham mice (41.62% vs. 59.29%; *p*-value < 0.001). In contrast, APPKI-*SynCav1* exhibited significantly more frequent freezing behavior versus APPKI-Sham (50.03% vs. 41.62%; *p*-value = 0.01) (Fig. 4d), indicating that *SynCav1* preserved hippocampal-dependent contextual memory recall in APPKI mice. On Day 3, both APPKI-Sham and APPKI-*SynCav1* groups displayed significantly fewer freezing behavior versus the WT-Sham group, indicating deficits in non-hippocampal-dependent cue memory (Fig. 4e), an expected result since cued memory involves multiple brain regions, such as the amygdala and prefrontal cortex, that did not receive *SynCav1*.

Given that sex is a crucial variable in the AD-affected patient population, we also assessed the effect of *SynCav1* therapy in female APPKI mice. Both APPKI-Sham and APPKI-*SynCav1* groups exhibited decreased freezing on Day 1 (Fig. 4f), consistent with what was observed in male APPKI cohorts. On Day 2, female APPKI-*SynCav1* mice displayed significantly greater contextual freezing behavior versus APPKI-Sham mice (51.16% vs. 37.06%; *p* = 0.03), demonstrating preserved hippocampal-dependent contextual memory recall (Fig. 4g). On Day 3, both APPKI groups displayed significantly fewer freezing behavior compared to the WT-Sham group (Fig. 4h).

OF test was performed to evaluate general locomotion and anxiety-like behavior. In the male cohorts, both APPKI-Sham and APPKI-*SynCav1* mice exhibited significantly lower moving distance and velocity compared to the WT-Sham group; no significant difference was observed between APPKI-Sham and APPKI-*SynCav1* (Fig. 4i). No significant difference was detected in time spent in arena center among all three groups. In the female cohorts, no difference in total distance traveled, velocity, and time spent in the arena center across all three groups (Fig. 4j). Overall, we have demonstrated that *SynCav1* hippocampal delivery consistently preserved hippocampal-dependent contextual memory recall in two AD transgenic animal models without inducing side effects on general motor function and anxiety-related behaviors.

### *SynCav1* increased p-CaMKII and p-CREB expression in primary cortical neurons

**Fig. 5:**
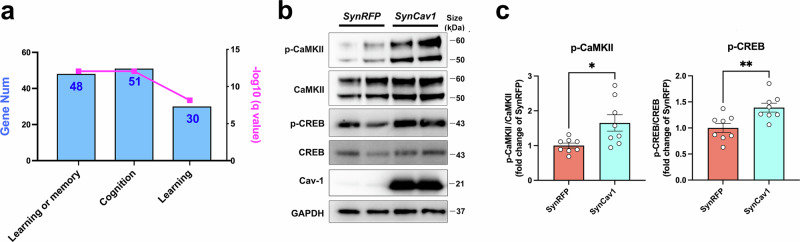
*SynCav1* increases p-CaMKII and p-CREB expression in primary mouse cortical neurons. **a** The enrichment of GO for biological processes related to “learning or memory,” “cognition,” and “learning.” Specific gene numbers are indicated on each bar graph. **b**, **c** IB of primary cortical neuron culture for p-CaMKII, CaMKII, p-CREB, CREB, Cav-1, GAPDH with quantification (**c**). Data are presented as mean ± SEM and analyzed using the Student’s *t* test. *n* = 8 (technical duplicates from four independent experiment). Significance was assumed when *p* < 0.05. *p < 0.05, **p < 0.01

In consideration of the robust cognitive functional improvement and upregulation in learning or memory-related pathways in the AD-*SynCav1* group relative to AD group as identified by enrichment of GO for biological processes (Fig. 5a), we further assessed the effect of *SynCav1* on synaptic plasticity signaling using primary cortical neurons. IB revealed that the expression of phosphorylated calcium/calmodulin kinase II (p-CaMKII) and phosphorylated cyclic-AMP response element-binding protein (p-CREB) (Fig. 5b, c), two molecular signatures of neuronal activity known to modulate synaptic plasticity and contribute to long-term memory formation, was significantly upregulated in *SynCav1*-transfected neurons compared to *SynRFP*-transfected control neurons.

### *SynCav1* preserved the expression of activity-dependent neuroprotective protein (ADNP)

**Fig. 6:**
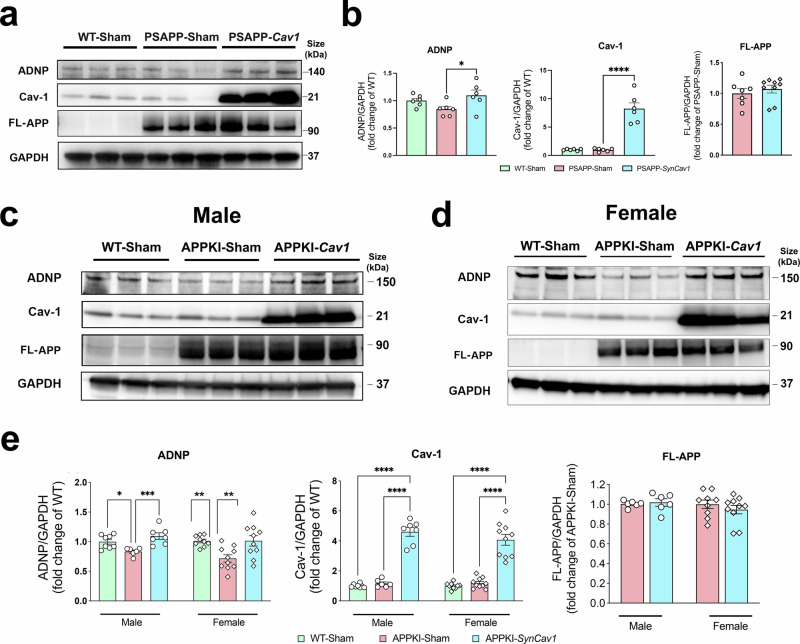
*SynCav1* maintains hippocampal ADNP expression in PSAPP and APPKI mice. **a**, **b** IB of hippocampal homogenates for ADNP, Cav-1, and Fl-APP for PSAPP mice and quantification. *n* = 6–8. **c–e** IB of hippocampal homogenates for ADNP, Cav-1, and Fl-APP for male (*n* = 6–8) and female (*n* = 9–10) APPKI mice with quantification. Data are presented as mean ± SEM and analyzed using One-way ANOVA or Student’s *t* test within each sex. Significance was assumed when *p* < 0.05. *p < 0.05, **p < 0.01, ***p < 0.005, ****p < 0.0001

Further analysis of enrichment of GO pathways involved in learning, memory, and cognition revealed ADNP as one of the genes with priority ranking in Hierarchical clustering analysis. Since ADNP has been shown to afford neuroprotective effects via stabilizing microtubules, protecting axonal transport, and attenuating oxidative stress,^21^ we thus validated the expression of ADNP in hippocampal tissue by IB. *SynCav1* injection raised hippocampal Cav-1 expression by 8-fold (Fig. 6a, b). Compared to PSAPP-Sham mice, a significantly higher ADNP protein expression was detected in the hippocampus of PSAPP-*SynCav1* mice, results consistent with findings from RNA sequence analysis. A downward trend (*p* = 0.12) in ADNP expression, albeit not significant, was observed in PSAPP-Sham mice compared to WT mice. No significant difference was found in full-length (FL) APP between PSAPP-Sham and PSAPP-*SynCav1* mice.

In APPKI mice, *SynCav1* injection resulted in an approximately 5-fold increase in hippocampal Cav-1 expression (Fig. 6c–e). Similar to what was observed in PSAPP mice, while a significant decrease in ADNP expression was detected in the hippocampus of both male and female APPKI-Sham mice compared to the WT-Sham group, APPKI-*SynCav1* mice exhibited significantly higher ADNP protein expression compared to APPKI-Sham mice, confirming that *SynCav1* preserved ADNP expression in an additional AD mouse model. No significant change in FL-APP expression was detected between APPKI-Sham and APPKI-*SynCav1* groups.

### *SynCav1* preserved ADNP expression by maintaining MLR-localized PAC1R expression

**Fig. 7:**
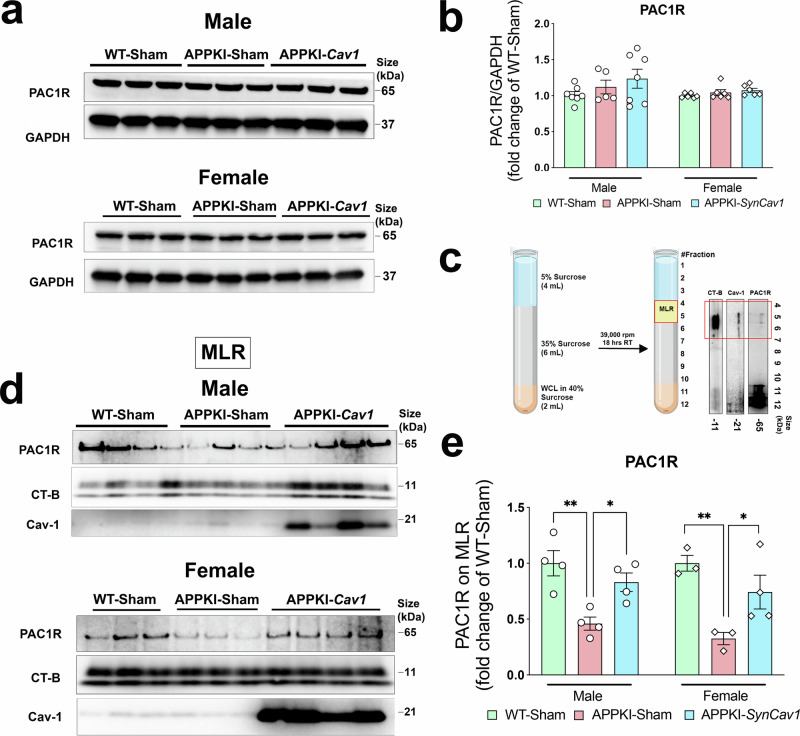
*SynCav1* maintains ADNP expression in male and female APPKI mice by preserving MLR-localized PACAP/PAC1R signaling. **a**, **b** IB of hippocampal homogenates of PAC1R expression with quantification. *n* = 5–7. **c** Schematic for separation of MLRs using sucrose density gradient fractionation. MLRs are concentrated to fractions 4–6 (boxed) at the 5% and 35% interface of the density gradient. **d**, **e** Immunoblot of MLRs-containing buoyant fractions (pulled from layers 4–6) for PAC1R, CT-B, and Cav-1 in male (*n* = 4) and female (*n* = 3) APPKI mice with quantification. Data are presented as mean ± SEM and analyzed using one-way ANOVA within each sex. Significance was assumed when *p* < 0.05, *p < 0.05, **p < 0.01

Since ADNP expression could be upregulated by PAC1R activation by pituitary adenylate cyclase-activating peptide (PACAP), and the activation of this signaling pathway has been shown to exert neuroprotective effects in cerebral ischemia and hemorrhagic injury,^22–24^ we examined PAC1R expression in hippocampal homogenates and MLRs-containing subcellular fractions. No significant difference in PAC1R expression was detected at the whole-cell level among all three groups (Fig. 7a, b). However, IB of the MLRs-containing fractions revealed a significantly greater expression of PAC1R in the APPKI-*SynCav1* group compared to the APPKI-Sham group, which showed significantly decreased PAC1R expression in the MLRs versus the WT-Sham group (Fig. 7c–e). These findings imply that the WT-level ADNP expression in *SynCav1*-treated mice was likely due to the preservation of PACAP/PAC1R signaling.

## Discussion

In this study, we assessed the therapeutic effects of post-symptomatic hippocampal *SynCav1* delivery in two distinct AD rodent models. Building on our previous findings that demonstrated *SynCav1’s* neuroprotective effects in both aging and presymptomatic AD mouse models, we now present that a one-time *SynCav1* administration at symptomatic stages of AD preserved hippocampal-dependent memory in AD mice. Corresponding to preserved cognitive function, transcriptomic profiling revealed that *SynCav1*-treated AD mice exhibited an expression profile that was distinct from AD mice and was more similar to that of WT mice. GO enrichment analysis indicated multiple upregulated pro-cognition pathways and downregulated neurodegenerative pathways in *SynCav1*-treated AD mice compared to AD mice. Furthermore, based on the transcriptome analysis, we validated ADNP as a novel candidate mediating *SynCav1*’s protective effect on cognitive functions.

We initially delivered *SynCav1* to early symptomatic (6 m) PSAPP mice, which exhibited obvious learning deficits in the fear-conditioning test (Supplementary Fig. 1a–c) that were consistent with previously reported findings.^25^ FC test performed at 12 m revealed preserved hippocampal-dependent memory in PSAPP-*SynCav1* mice compared to PSAPP-Sham mice. Matching this observed better cognitive performance, transcriptome profiling showed that *SynCav1*-treated PSAPP mice exhibited a similar gene expression profile to that of healthy WT mice, indicating that *SynCav1* protects the hippocampus by counteracting AD-induced pathological changes at the transcriptional level. Moreover, GO enrichment analyses indicated downregulation in multiple neurodegenerative disease-related pathways (AD, PD, MS, and ALS) and upregulation in memory, cognition, and synaptic function and activity-related pathways. These findings suggest that the neuroprotective effect of *SynCav1* may be attributed to its ability to maintain neuronal activity and synaptic plasticity. To investigate this possibility, we examined the effect of *SynCav1* on p-CaMKII and p-CREB expression, two molecular signaling components related to neuronal activity and synaptic function, using mouse primary cortical neurons. Our findings revealed that *SynCav1*-transfected neurons exhibit enhanced phosphorylation of CaMKII (Thr-286) and CREB (Ser-133), both of which are downregulated in postmortem brains from AD patients and transgenic animal models.^26–28^ The temporal kinetics of CaMKII and CREB phosphorylation (i.e., activation) are transient cellular events,^29–32^ thus making it difficult to detect changes in expression of these signaling pathways in brain tissue in vivo several months after *SynCav1* delivery. This is why the current study utilized primary neurons in vitro to capture potential transient cell signaling events. These findings strongly support our hypothesis that *SynCav1*, in part, protects the hippocampus from AD-associated neuropathological changes via enhancing molecular signaling components linked to synaptic plasticity, neuronal activity, and neuroprotection (i.e., p-CaMKII, p-CREB). While these findings indicate the protective effect of *SynCav1* in symptomatic AD mouse model, PSAPP mice at 6 m exhibited neither astrogliosis (Supplementary Fig. 1d, f) nor amyloid plaque deposits,^25^ suggestive of an early symptomatic disease stage. Thus, the second part of the study tested whether *SynCav1* could afford similar neuroprotective effects in symptomatic APPKI animals. Once again, the neuroprotective effect of *SynCav1* on hippocampal-dependent memory was observed in APPKI mice without any changes in general behavior and anxiety-like behavior, further supporting the safety of *SynCav1* treatment in AD.

ADNP is tightly associated with synaptic plasticity and neuronal integrity, functioning as a key regulator for cognition.^33,34^ A gradual decrease in cortical ADNP expression has been previously reported in the FTD mouse model.^35^ Downregulation of ADNP was similarly reported from a clinical study examining serum collected from symptomatic AD patients, linking decreased ADNP expression to disease progression.^36^ In the current study, ADNP was identified as one of the top DEGs related to many biological pathways, including memory, synaptic activity, and microtubule bundle formation. Interestingly, research from other labs showed that ADNP protects glutamatergic synapses^37^ and stabilizes microtubules via tau-microtubule binding,^38–40^ consistent with pathways identified by the transcriptome. Importantly, we confirmed decreased ADNP in the hippocampus of Sham-surgerized APPKI mice and preserved ADNP expression in *SynCav1*-treated PSAPP and APPKI mice. ADNP expression can be regulated by the PACAP/PAC1R signaling pathway, a peptide-G protein-coupled receptor (GPCR) system that regulates synaptic function and neurite outgrowth.^22,41–43^ PAC1R activation has been shown to occur through Cav-1-enriched microdomains (i.e., MLRs),^44,45^ we thus further analyzed MLR-localized PAC1R expression. Our result showed a significant loss of MLRs-localized PAC1Rs in APPKI-Sham mice, which was in contrast to preserved MLRs-localized PAC1Rs in APPKI-*SynCav1* mice. These findings suggest that neuronal Cav-1 and Cav-1-associated MLRs may provide a neuroprotective signaling axis involving PAC1R-ADNP in AD. Future studies are needed to test whether inhibition of PAC1R and/or ADNP interferes with the cognitive benefits afforded by *SynCav1* gene delivery.

Ample evidence shows that APP cleavage by β- and γ-secretase occurs in MLR domains,^46,47^ and MLRs play an important role in the cellular uptake and transmission of Aβ. It is plausible that *SynCav1*’s neuroprotective effect may be mediated through modification of MLR-localized protein complexes. However, analysis of amyloid plaque burden showed no significant changes between *SynRFP* and *SynCav1*-treated PSAPP and APPKI mice (Supplementary Fig. 2), indicating that *SynCav1*’s neuroprotective and therapeutic effect is not through decreasing toxic amyloid-related peptide species. A limitation to the current study is that *SynCav1* expression was restricted to the hippocampus, which constrains the ability to combat global brain atrophy exhibited in late-stage AD. To address the issue of limited CNS transgene biodistribution, future studies are needed to explore whether achieving greater biodistribution of *SynCav1* in the CNS can further enhance its neuroprotective efficacy and therapeutic potential.

In summary, *SynCav1* demonstrated a promising effect in preserving cognitive function despite being given at the symptomatic stage. While multiple newly FDA-approved treatments focus on targeting Aβ clearance in AD patients, the therapeutic value of *SynCav1* lies in its ability to protect vulnerable neurons and augment cellular responses to either endogenous or applied exogenous neuroprotective factors (i.e., select receptor agonists), mechanisms that differ from currently approved therapies. Due to the multitude of neurotoxicity in the AD brain, further studies are warranted to investigate *SynCav1*’s therapeutic role when combined with amyloid-targeted drugs to enhance clinical outcomes.

## Materials and methods

### Animals

All mice (PSAPP-transgenic (Tg) (APPSwePS1d9; The Jackson Laboratory, Bar Harbor, ME, USA, APPKI (*App*^*NL-G-F/NL-G-F*^, Riken Institute, Japan)) were treated in compliance with the Guide for the Care and Use of Laboratory Animals (National Institutes of Health, Bethesda, MD, USA). Transgene (Tg) negative mice from the PSAPP strain were used as controls (WT), and C57/BL6J mice were used as control for APPKI groups. Mice were housed under normal conditions with a sufficient supply of water and food. PSAPP mice at 6 months of age (6 m) were selected for *SynCav1* delivery, considering that impaired long-term memory was already detected by this age both in our hand (Supplementary Fig. 1a–c) and other groups.^25^ Tg negative and PSAPP mice were allocated to 3 groups randomly: WT-Sham, PSAPP-Sham, and PSAPP-*SynCav1* to receive either hippocampal stereotactic injections of *AAV9*-*SynCav1* viral vector or sham surgery. Sham surgery involves creating needle paths using the same coordinates at 6 m. For transcriptome analyses, either *SynRFP* or *SynCav1* was delivered to the hippocampus to correct for potential AAV9 effects on viral infection (e.g., neuroinflammatory response). APPKI mice (8 m) were divided to 2 groups randomly: APPKI-Sham and APPKI-*SynCav1*; WT control mice (8 m) received sham surgery. At 12 m, open field (OF) and fear conditioning (FC) neurobehavioral tests were performed to evaluate general behavior and cognitive function, respectively, for both PSAPP and APPKI mice. At the conclusion of all behavior testing, mice were humanely euthanized, and the hippocampus was collected for biochemical and histological analysis (Fig. 1a). All animal use protocols (#20-030) were approved by the Veterans Administration San Diego Healthcare System Institutional Animal Care and Use Committee (IACUC) before procedures were performed.

### Stereotactic injection

Hippocampal delivery of AAV9-construct was performed on PSAPP (6 m) and APPKI mice (8 m). Briefly, mice were mounted onto a stereotaxic frame under anesthesia (2% isoflurane). Bilateral burr holes were made with a hand-held micro drill, and hippocampal injection was done using a 33-gauge, 10-μL gas tight syringe (Hamilton, Reno, Nevada) controlled by Injectomate (RWD, Shenzhen, China). A total of 3 μL of adeno-associated virus serotype 9 (AAV9) (viral titer: 2 × 10^10^ genome copies (g.c.)/μL) containing *SynCav1* was injected bilaterally into hippocampal region over 180 seconds at three locations in each hemisphere (0.5 μL/site, 1st site: AP: 1.80 mm, Lat: 1.50 mm, DV: 1.7 mm; 2nd site: AP: 2.30 mm, Lat: 2.20 mm, DV: 1.75 mm; 3rd site: AP: 3.00 mm, Lat: 3.00 mm, DV: 3.00 mm) with 1 minute indwelling time (Fig. 1b). Sham surgery entails creating needle paths using the same coordinates.

### Behavior tests

#### Open field (OF)

The Open Field (OF) test was performed to assess spontaneous motor activity and anxiety-like behavior.^20^ Mice were transferred to the testing room and allowed to acclimate for 30 min before the test session. During the test session, mice were placed in a square arena (41 × 41 × 34 cm enclosures) illuminated by a bright light. The apparatus was cleaned between each animal. A computerized video tracking system (Noldus XT 7.1, Leesburg, VA, USA) was used to track and analyze their activities during a 10-min test session. Distance moved (cm), velocity (cm/s), and time spent in the center of the arena (s) were recorded to evaluate general and anxiety-like activity as previously described.

#### Fear conditioning (FC)

To determine whether *SynCav1* delivery at symptomatic age (6 m for PSAPP; 8 m for APPKI) could alleviate or delay cognitive decline in AD mouse models, fear conditioning (FC) was performed as previously described.^14^ Briefly, two distinct contexts were employed on different days to measure context-dependent memory and cued memory, respectively. On Day 1, mice were placed in the first test arena and presented with unconditioned stimuli (US: electric foot-shock) and conditioned stimuli (CS: auditory tone) controlled by a computer (Med Associates Inc., St. Albans, Vermont). A total of five tone-shock pairs were given at a varying interval of 30–90 s. On Day 2, mice were placed in day 1 test arena to assess hippocampal-dependent contextual memory recall. On Day 3, mice were placed in a different test chamber with varied wall patterns, light settings, and odors. Five auditory tones were played to assess non-hippocampal-dependent cued memory recall. The freezing percentage of each component was recorded and analyzed via Video Freeze (Med Associates Inc.; San Diego Instruments, San Diego, California). Mice that exhibited a high baseline freezing percentage on Day 1 (>10%) were excluded. Mice that failed to associate CS with US, as demonstrated by the lack of freezing behavior after five trials (freezing percent lower than 5% on Tone 5) on day 1 were also excluded from further analysis of memory performance on Day 2 and Day 3. Mice that exhibited high baseline freezing behavior (>15%) on Day 3 were excluded for analysis of cued memory recall to avoid confounding effects due to generalized fear.^48^

#### RNA extraction and purification

PSAPP mice that received either *SynRFP* (*n* = 3) or *SynCav1* (*n* = 4) injection and Tg-negative mice (*n* = 3) were perfused with ice-cold saline, and the hippocampal tissue was dissected and stored in −80 before RNA extraction. Total RNA was extracted using RNeasy lipid tissue kit (#74804, Qiagen) and assessed for quality using an Agilent Tapestation 4200. Samples with an RNA Integrity Number (RIN) greater than 8.0 were used to generate RNA sequencing libraries using the Illumina® Stranded mRNA Prep (Illumina, San Diego, CA). 500 ng of RNA was used for each sample and processed following the manufacturer’s instructions. Resulting libraries were multiplexed and sequenced with 100 base pair (bp) Paired End reads (PE100) to a depth of approximately 25 million reads per sample on an Illumina NovaSeq 6000. Samples were demultiplexed using bcl2fastq Conversion Software (Illumina, San Diego, CA).

#### RNA-seq processing

Software used for RNA-seq analyses is summarized in Bioinformatics Resources (Supplementary Table S1). FASTQ files were filtered to remove low-quality bases, TruSeq dual-index adapter sequences, and unpaired reads using *Trimmomatic*.^49^ Transcript-level mapping and quantification were performed by Salmon^50^ using the mouse transcriptome database (Gencode version M32). Flags *-seqBias* and *-gcBias* were applied during mapping to correct systematic biases commonly present in batch RNA-seq data. Quality of RNA-seq, mapping and quantification was assessed using *FastQC* (Babraham Bioinformatics, UK) and *MultiQC*^51^ tools.

#### Differential expression analysis

Gene-level quantification and annotation were done using *Tximeta* utility.^52^ Gene count matrices were normalized and differential expression analysis were done in *DESeq2*.^53^ Outlier samples were identified by Cook’s distance method and excluded. To enable comparative transcriptome analysis, gene counts were normalized using the variance stabilizing transformation (VST) algorithm^37,54^ to remove the dependence of the variance on the mean and to account for library size. The log_2_FC values were adjusted using the adaptive *t*-prior *apeglm* method.^55^ Significant DEGs were defined by log_2_FC, Wald test *P* < 0.05 and *P*_adj_ < 0.1 (adjusted *P* by Benjamini–Hochberg procedure in DESeq2). Batch effects were controlled using *removeBatchEffect*^19^ and *RUVseq*^56^ functions. DEGs were visualized using *PCAtools*, *ComplexHeatmap*, and *EnhancedVolcano* Bioconductor packages.

#### Gene set enrichment analysis

Biological interpretations of signaling pathways and GO terms were conducted using in cluster Profiler^57^ based on significance scores (*q*-values < 0.05) calculated by Fisher exact test^57^ adjusted by Benjamini-Hochberg procedure. KEGG^58^ and GO public databases^59^ were used for data mining. Data were visualized in Bioconductor^60^ and R.

#### In vitro primary neuron experiments

Primary cortical neurons were isolated from embryonic day 19 C57BL/6J wild-type mice using the papain dissociation system (#130-092-628, Miltenyi biotec). Neurons were cultured in Neurobasal media supplemented with B27 (2%), N2 (1%), 250 mM GlutMAX1, and penicillin/streptomycin (1%) and grown on poly-L-ornithine/laminin-coated plates at 37 °C in 5% CO_2_. Neurons were treated with lentivirus containing either *SynCav1* or *SynRFP* construct (8 MOI) on day 7 for 48 h. On day 11, cells were harvested with cold high-pH lysis buffer (500 mM sodium carbonate; pH 11.0) containing protease and phosphatase inhibitor cocktail. Cell Lysates were then sonicated for 3 times on ice prior to immunoblot with p-CREB (Ser133) (Cell Signaling #9198; 1:1500), CREB (Cell Signaling #9197; 1:1000), p-CaMKII (Cell Signaling #12716; 1:1500), CaMKII (Cell Signaling #3362; 1:1000), Cav-1 (Cell signaling technology #3267; 1: 1000), and GAPDH (Cell signaling #2118S; 1: 2000). Technical duplicates are used in each experiment and four independent experiments were performed.

#### Immunoblot (IB)

Freshly dissected hippocampi were homogenized in cold high-pH lysis buffer and sonicated three times for 10 s on ice. The Bradford assay was used to determine and standardize sample concentration. Tissue homogenates were then immunoblotted (IB) with primary antibodies for Cav-1 (Cell signaling technology #3267; 1: 1000), APP (#Biolegend #39320, 1:1000), GAPDH (Cell signaling #2118S; 1: 2000), ADNP (Proteintech # 17987-1-AP, 1:400), and PAC1R (ThermoFisher # PA5-96229, 1:1000) overnight at 4 °C followed by incubation with HRP-linked secondary antibody (Cell signaling technology # 7074) for 1 hour in room temperature. Signal was visualized with Lumigen UCL Ultra (TMA-6, Lumigen, USA) followed by densitometric analysis using Image J.

#### Biochemical characterization of MLRs

Hippocampal tissues homogenized in cold 500 mM sodium carbonate buffer were subjected to sucrose density gradient fractionation as previously described.^14^ Briefly, tissue lysate was normalized to 0.8 mg/ml. Next, 1 mL of lysate was mixed with an equal volume of 80% sucrose solution in MES-buffered saline (25 mM MES, 150 mM NaCl, 2 mM EDTA, pH 6.5) to generate a 40% sample/sucrose mixture. Subsequently, 6 mL of 35% and 4 mL of 5% sucrose solution were layered gently on top of the 40% mixture. Lastly, the sucrose density gradient was ultracentrifuged using a SW-41 rotor at 39,000 rpm for 18 h at 4 °C. On the following day, layer 4–12 fractions (1 mL/fraction) were sequentially collected for IB. Buoyant fractions (layers 4–6) that contain the MLRs are concentrated to 5%/35% sucrose gradient interface. Cholera toxin subunit B (Invitrogen # v34405) and anti-CTB antibody (Invitrogen # MA1-83519, 1:1000) were used to visualize the MLRs in buoyant fractions.

#### Immunofluorescence microscopy

Tissue sections are washed 3x in TBS for 10 min each. Next, antigen retrieval was performed using pH 6 10 mM citrate buffer with 0.05% Tween at 80 °C for 20 min. At the end of the antigen retrieval process, tissue sections are allowed to cool down for 10–15 min. Finally, two additional TBS washes are performed for 5 min each. Floating sections were then blocked with 5% goat serum and incubated with rabbit anti-Cav-1 (Cell Signaling technology #3267, 1:1000) at 4 °C overnight. Slices were then incubated with species-specific fluorescent secondary antibodies in the dark for 1 h and preserved with anti-fade DAPI-mounting medium.

For amyloid-β plaque staining, sections were incubated in 98% formic acid for 5 min to fully expose the epitope before blocking. Alex fluor 488 anti-β-Amyloid 1–16 antibody (BioLegend # 39320, 1:200,) was used to visualize amyloid plaques. Plaque was traced by ImageJ to generate plaque area for further analysis. All images are captured by Keyence All in One microscope (Keyence, Japan).

### Statistical analysis

All data were analyzed using GraphPad Prism 8 (GraphPad Software, La Jolla, California, USA). For comparison between two groups, data was checked for normal distribution and analyzed by either Student’s *t* test. For comparison between three groups, data was analyzed using either One-way ANOVA or Two-way ANOVA followed by Bonferroni’s post hoc analysis or LSD multiple post hoc analysis as described. All data is presented as mean ± standard error of the mean (SEM), group size for each independent experiment was described in the figure legend and significance was assumed when *p* < 0.05. Experimental groups were blinded to the observer, and the code was broken for final analysis.

## Supplementary information

Supplemental Materials

Supplementary Table S2. Differentially expressed genes

Supplementary Table S3 KEGG pathways

Supplementary Table S4. GO Biological processes

Raw Data for Immunoblots

## Data Availability

The original and normalized transcriptomics data are available in the Gene Expression Omnibus (GEO) repositories (GSE278571). DEGs and respective statistical parameters are included in Supplementary Data S2. Enrichment analysis results are included in Supplementary Data S3 and S4.
