# Supplementary material for: Neuron-targeted caveolin-1 overexpression attenuates cognitive loss and pathological transcriptome changes in symptomatic Alzheimer’s disease models
Source: Signal Transduct Target Ther. 2025 May 28;10:172. doi: 10.1038/s41392-025-02258-z (PMC12117040; doi:10.1038/s41392-025-02258-z)
Supplement: Supplementary file 1 — Supplemental Materials [file 41392_2025_2258_MOESM1_ESM.docx]

Supplementary Materials for

Neuron-targeted caveolin-1 overexpression attenuates cognitive loss and pathological transcriptome changes in symptomatic Alzheimer’s disease models

**Authors:** Dongsheng Wang^1,2^, Andrei V. Chernov^1,2^, Ryan Lam^1,2^, Hongxia Wang^1,2^, Wenxi Li^1,2^, Xiaojing Li^1,2^, Tiffany Duong^1,2^, Shanshan Wang^1,2*^, Brian P. Head^1,2*^

Correspondence to: shw049@health.ucsd.edu; bhead@health.ucsd.edu

**This PDF file includes:**

Figures. S1 to S2

Tables S1

**Other Supplementary Materials for this manuscript include the following:**

Three additional supplementary tables (Supplementary Tables 2–4) are provided as separate files due to their detailed content.

Table S2: Differentially Expressed Genes

Table S3: KEGG Pathways

Table S4: GO Biological Processes

Figure. S1.

**
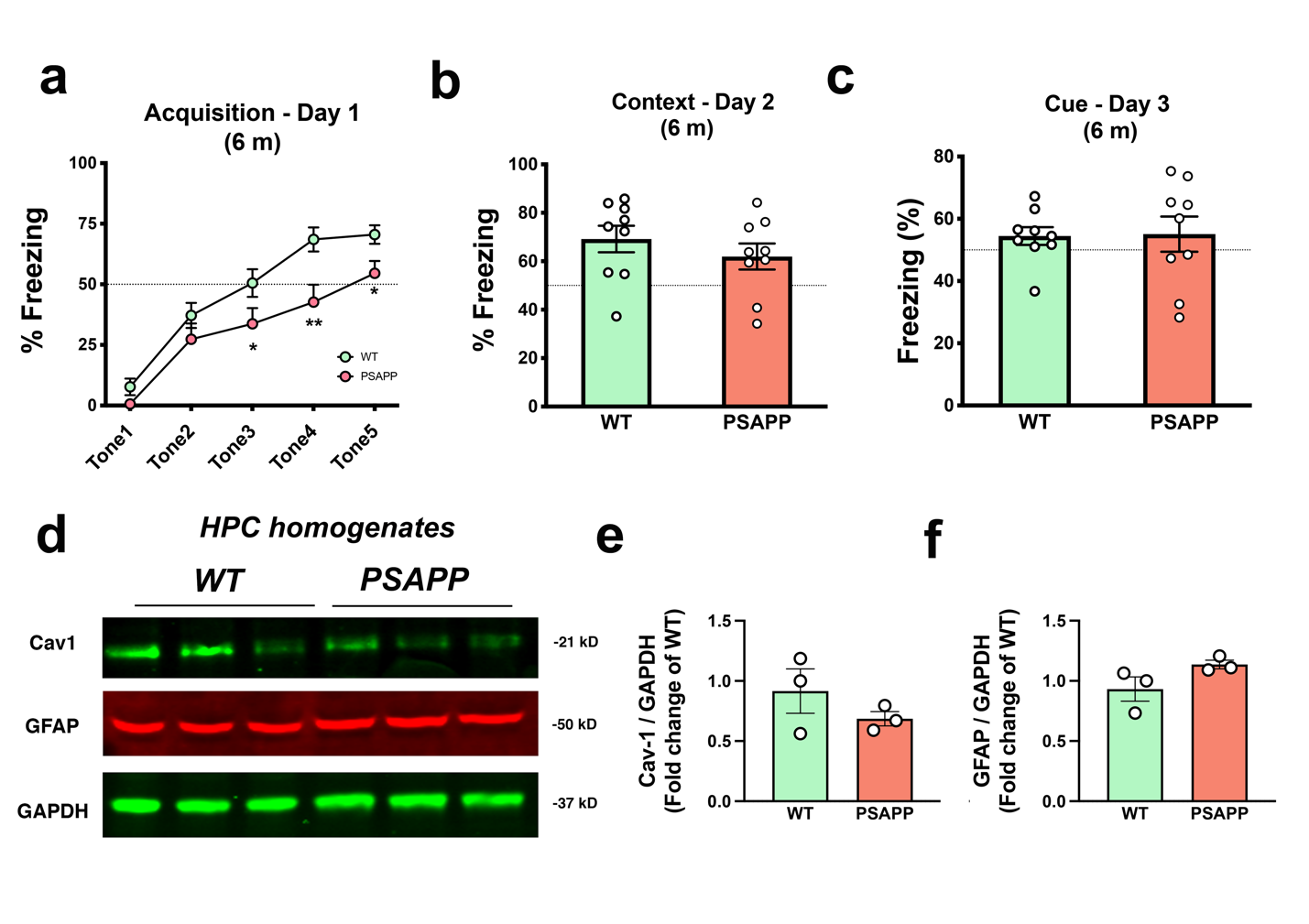
**

**Supplemental Figure 1.** Characterization of 6-month-old early symptomatic PSAPP mice. **a-c** Fear conditioning test revealed a significant learning deficit (Day 1) at 6 m. No difference was detected in contextual memory and cue memory. Data (*n* = 9 per group) are presented as percent (%) freezing mean ± SEM. Day 1 data were analyzed by using two-way analysis of variance (ANOVA) followed by Fisher’s LSD multiple comparison test. Day 2 and Day 3 data were analyzed using unpaired student’s t test. **d-f** Immunoblot of hippocampal tissue (*n* = 3 per group) detects no difference in GFAP and Cav-1 expression between control mice and 6-month-old PSAPP mice. IB data were analyzed using unpaired student’s t test. Significance was assumed when p < 0.05. *p < 0.05, **p < 0.01.

Figure. S2.

**
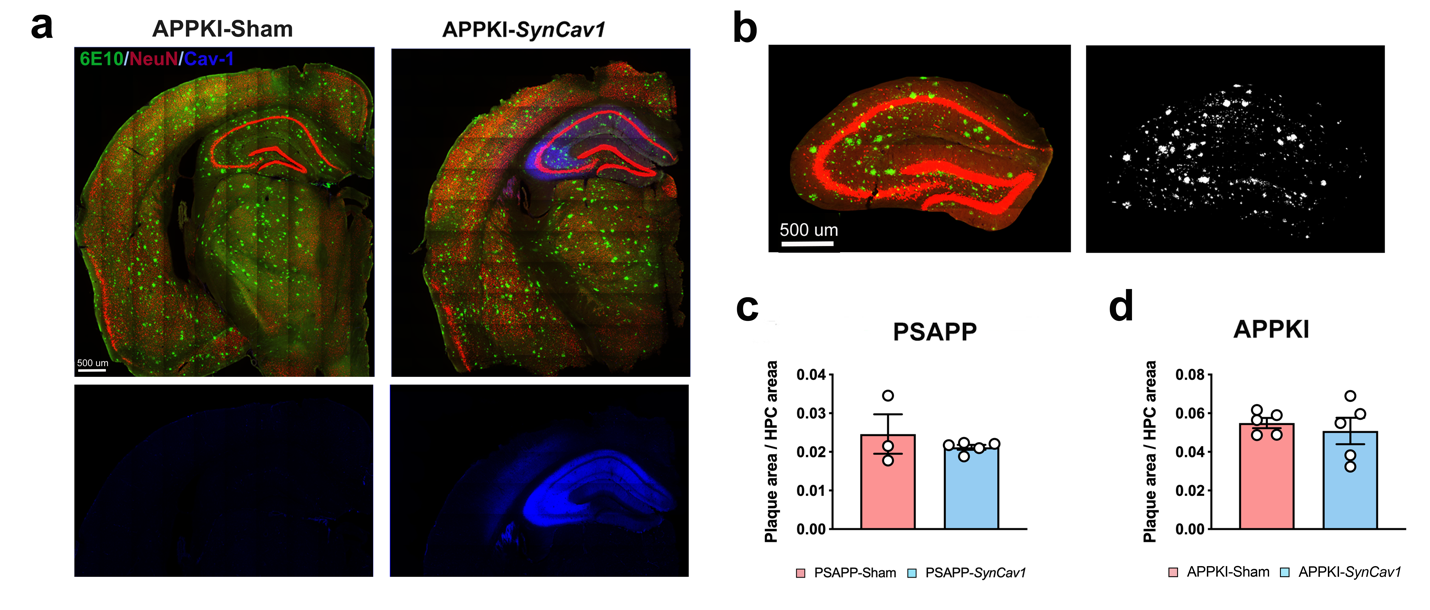
Supplemental Figure 2.** Hippocampal *SynCav1* delivery did not affect amyloid plaque pathology. **a** Representative IF image of APPKI-Sham and APPKI-*SynCav1* brain sections co-stained with NeuN (red), 6E10 (green), and Cav-1 (blue). Scale bar = 500 μm. **b** Enlarged image of the hippocampus (left). 6E10 channel is isolated for plaque area analysis (right). Scale bar = 500 μm. Quantitative analysis of total plaque area per hippocampus for (**c**) PSAPP mice (*n* = 3 for PSAPP-Sham; *n* = 5 for PSAPP-*SynCav1*) and (**d**) APPKI mice (*n* = 5 for APPKI-Sham; *n* = 5 for PSAPP-*SynCav1*). Data are presented as mean ± SEM. Data were analyzed using student’s t-test. Significance was assumed when p < 0.05.

**Table S1. Bioinformatic Resources**

| Description | Source | Identifier | URL |
| --- | --- | --- | --- |
| Trimmomatic | [2] | Version 0.39 | http://www.usadellab.org/cms/?page=trimmomatic |
| Tximeta | [3] | Version 3.11 | https://www.bioconductor.org/packages/release/bioc/html/tximeta.html |
| FastQC | Babraham Bioinformatics | Version 0.12.0 | https://www.bioinformatics.babraham.ac.uk/projects/fastqc/ |
| MultiQC | Stockholm University, Stockholm, Sweden [4] | Version 1.9 | https://multiqc.info/docs/ |
| DESeq2 | [5] | Version 1.36.0 | https://www.bioconductor.org/packages/devel/bioc/vignettes/DESeq2/inst/doc/DESeq2.html |
| Mouse genome | NCBI | Gencode M32 | https://www.gencodegenes.org/mouse/release_M32.html |
| clusterProfiler Bioconductor package | [6] | Release 4.8.1 | https://bioconductor.org/packages/release/bioc/html/clusterProfiler.html |
| Kyoto Encyclopedia of Genes and Genomes (KEGG) | [7] | Release 106.0 | https://www.kegg.jp/ |
| Gene Ontology (GO) | [8] | release 2023-05-10 | http://geneontology.org/ |
| R programming language |  | Version 4.3.1 | https://www.r-project.org/ |
| Bioconductor | [9] | Version 3.17 | https://bioconductor.org/ |
| SRplot Heatmap | [1] |  | https://www.bioinformatics.com.cn/plot_basic_cluster_heatmap_plot_024_en |

**REFERENCES**

1. Tang, D., et al., SRplot: A free online platform for data visualization and graphing*.* *PLoS One*. **18**, e0294236 (2023).

2. Bolger, A.M., M. Lohse, and B. Usadel, Trimmomatic: a flexible trimmer for Illumina sequence data*.* *Bioinformatics*. **30**, 2114-2120 (2014).

3. Love, M.I., et al., Tximeta: Reference sequence checksums for provenance identification in RNA-seq*.* *PLoS Comput Biol*. **16**, e1007664 (2020).

4. Ewels, P., et al., MultiQC: summarize analysis results for multiple tools and samples in a single report*.* *Bioinformatics*. **32,** 3047-3048 (2016).

5. Love, M.I., W. Huber, and S. Anders, Moderated estimation of fold change and dispersion for RNA-seq data with DESeq2*.* *Genome Biol*. **15**, 550 (2014).

6. Wu, T., et al., clusterProfiler 4.0: A universal enrichment tool for interpreting omics data*.* *Innovation (Camb)*. **2**, 100141 (2021).

7. Kanehisa, M., et al., KEGG for taxonomy-based analysis of pathways and genomes. *Nucleic Acids Res*. **51**, D587-D592 (2023).

8. Ashburner, M., et al., Gene ontology: tool for the unification of biology*.* The Gene Ontology Consortium*.* *Nat Genet*. **25**, 25-29 (2000).

9. Gentleman, R.C., et al., Bioconductor: open software development for computational biology and bioinformatics*.* *Genome Biol*. **5**, R80 (2004).
